# Lumbar Degenerative Disease Part 1: Anatomy and Pathophysiology of Intervertebral Discogenic Pain and Radiofrequency Ablation of Basivertebral and Sinuvertebral Nerve Treatment for Chronic Discogenic Back Pain: A Prospective Case Series and Review of Literature

**DOI:** 10.3390/ijms21041483

**Published:** 2020-02-21

**Authors:** Hyeun Sung Kim, Pang Hung Wu, Il-Tae Jang

**Affiliations:** 1 Nanoori Gangnam Hospital, Seoul, Spine Surgery, Seoul 06048, Korea; pang_hung_wu@nuhs.edu.sg (P.H.W.); nanooriresearch@gmail.com (I.-T.J.); 2 National University Health Systems, Juronghealth Campus, Orthopaedic Surgery, Singapore 609606, Singapore

**Keywords:** degenerative disc disease, lumbar spondylosis, pain management, sinuvertebral nerve, basivertebral nerve, discogenic back pain, pathophysiology of back pain, endoscopic spine surgery, radiofrequency ablation, prolapsed intervertebral disc

## Abstract

Degenerative disc disease is a leading cause of chronic back pain in the aging population in the world. Sinuvertebral nerve and basivertebral nerve are postulated to be associated with the pain pathway as a result of neurotization. Our goal is to perform a prospective study using radiofrequency ablation on sinuvertebral nerve and basivertebral nerve; evaluating its short and long term effect on pain score, disability score and patients’ outcome. A review in literature is done on the pathoanatomy, pathophysiology and pain generation pathway in degenerative disc disease and chronic back pain. 30 patients with 38 levels of intervertebral disc presented with discogenic back pain with bulging degenerative intervertebral disc or spinal stenosis underwent Uniportal Full Endoscopic Radiofrequency Ablation application through either Transforaminal or Interlaminar Endoscopic Approaches. Their preoperative characteristics are recorded and prospective data was collected for Visualized Analogue Scale, Oswestry Disability Index and MacNab Criteria for pain were evaluated. There was statistically significant Visual Analogue Scale improvement from preoperative state at post-operative 1wk, 6 months and final follow up were 4.4 ± 1.0, 5.5 ± 1.2 and 5.7 ± 1.3, respectively, *p* < 0.0001. Oswestery Disability Index improvement from preoperative state at 1week, 6 months and final follow up were 45.8 ± 8.7, 50.4 ± 8.2 and 52.7 ± 10.3, *p* < 0.0001. MacNab criteria showed excellent outcomes in 17 cases, good outcomes in 11 cases and fair outcomes in 2 cases Sinuvertebral Nerve and Basivertebral Nerve Radiofrequency Ablation is effective in improving the patients’ pain, disability status and patient outcome in our study.

## 1. Introduction

Degenerative disc disease is a leading cause of chronic back pain in the aging population in the world [1]. Disc consists of nucleus pulposus, annulus fibrosus and adjacent cartilaginous end plates. Various factors had been studied to determine the cause of degenerative disc disease and its pain generation pathway in an attempt to find an answer to either reverse and prevent further deterioration of degeneration or provide an optimal pain management strategy to alleviate the pain and suffering for patients with degenerative disc disease [2,3,4,5]. Various genetics, environmental, biomechanics and anatomical variation are found to have association with degenerative disc disease. Pain generators are postulated to be discogenic origin, although recent studies found some association with adjacent vertebral end plates and vertebral bodies, known as modic changes. A normal disc is aneural and avascular, but pathological pain innervation pathways are generated by stimulation by inflammatory pathways with its secreted cytokines leading to an inflammatory response leading to neurotization of the diseased disc [6]. Sinuvertebral nerve and basivertebral nerve are associated with the pain pathway [7,8,9,10,11,12]. In this study, we perform a prospective study using radiofrequency ablation on sinuvertebral nerve and basivertebral nerve and evaluate its short and long term effect on pain score, disability score and patients’ outcome and discuss the pathoanatomy, pathophysiology and pain generation pathway in degenerative disc disease and chronic back pain.

## 2. Results

**Figure 1:**
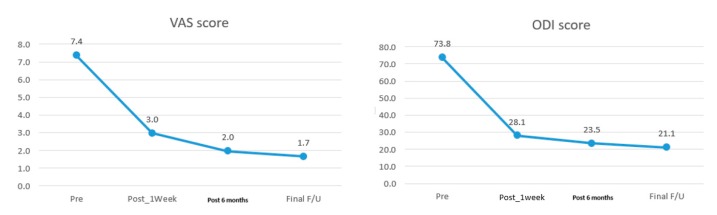
Mean Visual Analogue Scale Pre-operative, 1 week, 6 months post-operatively and Oswestry Disability Index at 1 week, 6 months post-operatively and final follow up showing clinically significant decrease in disability.

A total of 30 patients are included in the study who had undergone radiofrequency ablation procedure during the study period. Mean follow up was 14.9 (6–31) months with mean age of 48 (19–68) years old. They presents with a mean duration of 27 (8–130) months of discogenic back pain. 11 out of 30 of the cases are from L4/5 disc, 15 from L5/S1 and 4 cases had multiple levels of involvement. In 6 cases, Transforaminal Endoscopic Lumbar approach is used to perform radiofrequency application of which 5 of the cases are involving L4/5 degenerated disc protrusion, one case involved 2 levels i.e., T12/L1 and L3/4 of degenerative disc protrusions. In the transforaminal approach group 4 of the 6 cases used local anesthesia as a method of anesthesia while 2 patients had requested to be under regional anesthesia as the choice of anesthesia All except 2 of the other 24 cases were under regional anesthesia. In 8 cases, Interlaminar Endoscopy Lumbar to Disc Approach was performed with radiofrequency application, in all 8 cases, the diagnosis is degenerated disc protrusion of L5/S1 intervertebral disc with discogenic back pain on top of radicular leg pain. 16 cases of combined Lumbar Endoscopic Unilateral Laminotomy Bilateral Decompression with interlaminar radiofrequency ablation, all of the 16 cases has background mild spinal stenosis marked by thickening of ligamentum flavum with degenerated disc protrusion and predominant discogenic back pain and neuropathic leg pain but no spinal claudication. All except 2 patients had intraoperative buttock twitching when radiofrequency was applied. The 2 cases who did not have any intraoperative buttock twitching were both L4/5 prolapsed intervertebral intervertebral disc with isolated discogenic back pain. Radiofrequency was applied despite no Kim’s twitching observed in both cases. There were 28 cases had intraoperative buttock twitching and 2 cases had no intraoperative buttock twitching. Their clinical outcome was not as good as the other patients with intraoperative twitching. MacNab Score were fair for both of these cases and VAS score and ODI for pre-operative, 1 week, 6 months and final follow up in first patient was VAS (6,4,4,4), ODI (64,42,38,38) respectively; second patientwas VAS (8,4,3,4) and ODI (80,36,32,38) respectively both seemed to have inferior results compared to other patients who had twitched intraoperatively during radiofrequency ablation. Radiological data was analyzed with 6 showing Modic type 2 changes and 24 with Modic type 1 changes. The stenosis is mild in all cases of preoperative MRI in terms of Schizas grading, 25 cases had A1, and 4 cases had A3 and 1 case of A4 grade. Intraoperative endoscopic video evaluation showed there are 6 type 2 grade and, 24 grade 3 Neovascularization and Adhesion grading cases adjacent to the disc and pedicle at the region of sinuvertebral and basivertebral nerves. There was a statistical significant improvement in VAS score with mean and range of pre-operative, 1 week, 6 months and final follow up are 7.37 (6–8), 2.96 (2–4), 1.97 (1–4) and 1.67 (1–4) respectively (Figure 1). Using paired T test, VAS score mean difference between 1wk, 6 months and final follow up with pre-operative were 4.4 ± 1.0, 5.5 ± 1.2 and 5.7 ± 1.3 respectively, *p* < 0.0001. There was a statistical significant improvement in ODI score with mean and range of pre-operative, 1 week, 6 months and final follow up are 73.83 (62–86), 28.07 (20–42), 23.47 (18–38) and 21.13 (2–38) (Figure 1). The mean improvement of ODI at 1week, 6 months and final follow up compared with pre-operative values are 45.8 ± 8.7, 50.4 ± 8.2 and 52.7 ± 10.3, *p* < 0.0001. MacNab’s criteria showed excellent outcomes in 17 cases, good outcomes in 11 cases and fair outcomes in 2 cases. Overall the fair outcome and less clinical significant improvement in outcomes score were associated with no intraoperative Kim’s twitching when radiofrequency application is applied. There is one complication of post-operative foot drop, which spontaneously recovered after 3 months, one recurrence of symptoms with conservative management, no revision surgery for all the patients (Table 1).

## 3. Discussion

Degenerative disc disease is a leading cause of chronic back pain. As the society is aging, quality of life in the aging population is central to geopolitical and healthcare developments in preparing for an aging population. However, in terms of years lived with disability burden pattern there is similar pattern in 1990 to 2010 with lower back pain being most likely cause of musculoskeletal disability [1]. Various biopsychosocial factors lead to disability in chronic back pain [13,14]. Degenerative disc disease is a leading cause of lower back pain on a global scale [15]. Intervertebral disc (IVD) is an avascular and aneural structure yet various studies showed that a diseased IVD lead to significant disability. This review addresses the pathophysiology of IVD pain by understanding the different anatomical parts, biomechanical changes, the interaction of local and regional cells with molecular factors. We also discuss the inflammatory reactions and interactions with various environmental factors which involved in disc injury and eventual degeneration. Further elaboration is made on sympathetic nerve channel development of sinuvertebral and basivertebral nerve in intervertebral disc pain and how radiofrequency ablation can help in treatment of degenerative disc disease [8,11,12].

### 3.1. Anatomy of a Normal Disc

All vertebra except atlas and axis is separated by intervertebral disc (IVD), which is labelled by the 2 adjacent vertebrae which it separates in between, for example Lumbar 4/5 intervertebral disc lies between lumbar 4 and lumbar 5 vertebrae. IVD serves to provide load distribution as a shock absorber, dispersing the weight and impact sustained. It also served as a channel for nutrition flow between spinal cord and spine [16]. The total height of all discs made up of 20–30% of the total height of spine in a standing human. Their shape, structure and mechanical properties vary depending on the location, loads and stress-strain patterns encountered in the person daily requirements [17]. Adult intervertebral disc is avascular and aneural, the flow of nutrients are by passive diffusion from adjacent end plate vessels and pre-disc vessels reaching from the inner layer of the disc in centrifugal manner to outer layer of disc diffusing outwards [18].

Intervertebral Disc is made up of Nucleus Pulposus (NP), Annulus Fibrosis (AF) and the cartilage vertebral endplates. Outer layer of AF is attached to end plate and vertebral epiphysis by means of strong Sharpey fibers. While the central AF collagen fibers coalesce with nucleus to insert themselves obliquely into the cartilaginous end plate [19].

### 3.2. Structure of Annulus Fibrosus

Annulus Fibrosus is a structured concentric lamellae consisting of fibrocartilaginous tissue. It is generally referred to 2 sections, the inner and outer annulus fibrosus. The main composition consists of water (approximately 70% inner layer and 60% outer layer), collagens mainly type I and type II collagen of 15% inner and 7% outer dry weight) [18,20]. The structure of annulus runs in a multiple alternating crisscross layers alternating collagen with varying angles throughout its lamellae. All collagen is a triple helical structure with 3 polypeptide chains [21]. The collagen has anisotropic, nonlinear and inhomogeneous mechanical property which help to withstand complex load pattern and in particular radial forces [22]. The composition of collagen concentration increases from a lower concentration to higher concentration creating a smooth transition zone of soft and spongy nucleus pulposus to a stronger and stiff outer annulus [23]. In the annulus fibrosus, proteoglycans are glycosylated proteins found extracellular matrix. The main ones found in AF are aggrecan and versican promoting hydration and mechanical strength of the disc. The attached keratin sulphate and chondrointin sulphate attached to their protein core of glyocosaminoglycans providing the ability to aggregate hyaluronic acid to promote osmotic swelling pressure required for biomechanical loading, there are also other smaller quantity of proteoglycan implicated in fibrillary collagen assembly [24]. Overall with its unique structure AF is able to transfer and distribute load allowing radial bulge, tensile stress, rotation and compressive loading facilitating IVD joint mobility [20]. Cyclic loading tests showed resilience and stability of mechanical loading integrity of AF, allowing it to withstand more than 10000 cycles of stress magnitude of less than or equal to 45% of its ultimate tensile strength [25].

### 3.3. Structure of Nucleus Pulposus

Nucleus Pulposus is a gelatinous core of the disc surrounded by AF which keeps it from herniating into the spinal canal. It consists mainly type II collagen and elastin fibers in a random organized manner with 20% of the dry weight of the disc. Elastin fibers helped in maintaining the collagen organization and elastic properties under various loads. Elastin microfibrils forms a meshwork around a central elastin core sustaining its elasticity [26]. It has a low density of chondrocyte like cells of 5000/mm^3^ contained in a proteoglycan gel of 50% dry weight. Nucleus is a poorly compressible structure with 80% water helping it carrying out its role of load dispersion, shock absorption and maintenance of internal pressure [18,27]. Metabolically active chondrocyte like cells with Golgi Apparatus and Endoplasmic Reticulum synthesize and turnover a large volume of extracellular matrix components of collagen and proteoglycans maintaining a high compressive load through movement of the waters and ions in the matrix. They also maintain tissue homeostasis, play a role in the physio-chemical properties of cartilage-specific macromolecules, and prevent degenerative diseases like degenerative disc disease and osteoarthritis [28].

### 3.4. Structure of Vertebral End Plates

Vertebral endplates are adjacent to each IVD. It composed of an osseous and a cartilaginous components. Its main constituents are chondrocytes, proteoglycans and collagen mesh network. Although vertebra end plates has similar macromolecules in their extracellular matrix as NP and AF but the proportion is different, providing a higher mechanical properties than both AF and NP [29]. The paired end plates are comprised of hyaline cartilage [30]. It serves as an interphase between hard cortical bone of vertebra body and transiting to the annulus and nucleus via attachment of sharpey fibers. This helps to keep disc pressurized and prevents it from bulging into the soft cancellous trabecular bone of vertebra. Endplates are the strongest part of the IVD and failure often associated in the Vertebra body fracture prior to end plate fracture [18]. Vertebral end plates are vascularized and it plays a vital role in maintaining nutrition diffusion and hydration of the disc [31]. The thickness and porosity of end plates vary from central to peripheral region. Central portion of end plate is thinnest and less dense as compared to the periphery where subchondral bone growth starts [30]. Mechanical properties such as stiffness and strength increase as lumbar spine descend from L1 to S1 [32] End plates are usually stronger and stiffer than the vertebra body in withstanding higher loads protecting it from tensile and compressive damage which are the common modes of failure [33].

### 3.5. Pathoanatomy: Structural Changes to a Disc in a Pathological State

Central to its main changes is the necrosis of chondrocyte like cells in nucleus, it is a natural process with accelerating rate as per aging. Starting around 2% at birth to 50% in adulthood. With these cells necrosis, pathological state such as cartilage—collagen interphase degradation, syndesmophyte formation with abnormal bone growth at the adjacent vertebra and calcification occurs, stiffening the IVD joint [34].

Vertebra end plate porosity increases up from 50–130% with aging and disc degeneration, affecting its mechanical property [32].

Blood and Nervous supply of IVD changes during different stages of life. During early stages of skeletal development, blood and lymph vessels are in abundance in majority of the disc with exception of nucleus. As skeletal maturation occurs, blood and lymph vessels migrate towards AF and end plates up to 12months of age. Beyond 12months of age to 20 years of age which marked skeletal maturity, blood vessels start to recede from nucleus and inner annulus to only remaining in outer annulus and end plates [35]. After full recede process of the blood supply to outer annulus and end plate, the nutrition supply is relying mainly on diffusion which often is responsible for lower regenerative potential of tissue during aging [36]. There are 2 pathways that diffusion occurs from the blood vessels at disc margin: the cartilaginous endplate-nucleus pulposus pathway and the annulus fibrosus periphery pathway [37]. Impairment of either of these pathways can lead to different degenerative patterns with cartilaginous endplate-nucleus pulposus pathway affecting mainly nucleus pulposus and annulus fibrous pathway affecting the annulus fibrosus using 3D finite element model [2].

### 3.6. Types of Pathology in a Disc

According to lumbar disc nomenclature version 2.0, disc conditioned are broadly classified into Noramal, Congenital/developmental variation, Degenerative changes, Trauma, Inflammatory/Infection, Neoplasia and Miscellaneous paradiscal masses of uncertain origin [38]. The authors would focus on degeneration in this review. Degenerative changes are sub classified as annular fissures, degeneration and herniation.

Annular fissure is separation between the AF fibers or between AF fibers and the vertebral bone. There are 3 patterns in AF fissures, namely, concentric fissure, radial fissure (vertical or oblique) and transverse fissure which is a horizontal oriented radial fissure. Annular tear is synonymous as fissure but it is a term which gives a connotation to traumatic cause.

Degeneration includes terms such as dessication of the disc, fibrosis, narrowing of disc space and diffuse bulge of annulus beyond its normal confine, mucinous degeneration and intradiscal gas. The associated consequences of degenerated disc such as syndesmophyte formation, sclerosis of end plates and modic changes seen in MRI are spectrum of disc degeneration [39,40,41].

Herniation is categorized into focal displacement of disc material beyond the normal limits of IVD space. Disc material can be any constituent of the disc i.e., annulus, nucleus, apophyseal bone, end plate or combination of the above. The IVD normal margin is defined by Vertebra body end plates in cephalad caudal dimension and outer rim of vertebral ring apophyses excluding the osteophytes. Focal displacement refers to less than 25% of the dimension in 90 ° of periphery of the disc in axial plane. A more diffuse presence of disc tissue extension beyond the confined of normal IVD is term bulging of disc. Asymmetric bulging of disc greater than 25% of disc circumference can occur in deformity of spine but not considered as a form of herniation [42]. Disc herniation is subclassified into protrusion or extrusion depending on the shape of disc material. Protrusion is seen if the edge of disc material present outside IVD space is less than the distance between the base of the disc herniation. There is typically no migration in a disc protrusion type of herniation. Disc herniation is categorized as contained which is when the displaced disc is covered by annulus fibers or posterior longitudinal ligament which would appear to have smooth margin on axial computer tomography (CT) or magnetic resonance imaging (MRI), they are typically contained by a few fibers of superficial posterior annular fibers or posterior longitudinal ligament while uncontained disc occurs when such restrain from the fibers are lost [42,43]. Extrusion is defined when disc material distance from its edge to base in at least one plane is more than the edges of the base or when there is no continuity exists in between disc material and its base. The latter form of extrusion is often termed sequestration when the disc material is completely separated from the parent disc. The term migration is used when displacement of disc material is beyond the IVD margin from the site of extrusion [44]. Degenerated disc includes all normal and pathologic degenerative processes in the disc [38]. Degenerative disc disease (DDD) includes both annulus fibrosis and adjacent apophyses degeneration termed as “spondylosis deformans “ and nucleus pulposus and vertebral end plate degeneration known as “intervertebral osteochondrosis”. It had been suggested that the former is a consequence of normal aging while the latter is pathological but not necessarily symptomatic [38].

### 3.7. Pathophysiology: Etiology of Degenerative Disc Disease

Degenerative Disc Disease (DDD) is a result of complex interplay of structural, genetics, environmental, trauma, and aging. Non traumatic degeneration starts with progressive decrease in disc nutrient supplies and changes in the extracellular matrix composition as part of aging process. Over time such changes leads to disc being less capable of maintaining its function of load distribution and ECM turnover. There is lowering of pH and oxygen concentration due to these changes [37]. Calcification of end plates developed due to these changes which further led to decrease in nutrient flow and blood supply leading to further dysfunction of disc subjecting in response to microtrauma. Accumulation of microtrauma over time and propagates the mechanical damage manifesting as annular fissure and nucleus damage [45].

### 3.8. Potential Trigger Events

Several trigger events can lead to progression of DDD. The cause and effect of the trigger is not direct correlation. It is likely cumulative effect of several factors acting in sync to cause the gradual and progressive process of DDD. Several triggers are discussed in this review. Structural changes and biomechanics of spine and pelvis, trauma, genetics, smoking, nutrition deficiency plays important role in leading to DDD.

### 3.9. Structural and Biomechanics of Spine and Pelvis Affecting DDD

Alterations in the regular alignment of spine in one segment of the spine will leads to compensatory changes in another part of spine to compensate the alignment in order for man to stay upright and look straight. Pelvis driven Lumbar disc disease is a possible mechanism of degenerative disc disease. Study showed that because pelvic morphology plays a key role defining the spinal shape as well as its load and function it thereby potentially predisposes the development of spinal degeneration if the shape of pelvis is out of normal range [46]. Vertebra body shape plays a role in determination of the stress distribution between the superior and inferior adjacent IVD. For example a wedge shape L5 will have different forces going through the L4/5 and L5/S1 disc compared to regular and rectangular shape L5. Study showed that asymmetry of superior and inferior endplates in the mid-sagittal plane is a risk factor for lumbar disc degeneration [47].

Fei et al., studied sagittal parameters and the effects on lumbar herniation. The author showed that the mean lumbar lordosis, sacral slope and thoracic kyphosis were significantly lower, with a higher pelvic tilt and the increased sagittal vertical axis in lumbar disc herniation group as compared to the control group in a young sample population [48]. Overall, vicious cycle situation where coronal and sagittal imbalance accelerated disc degeneration and advanced disc degeneration leads to subluxation of facet joints and abnormal weight bearing causing a deformity in the spine [49].

### 3.10. Muscle Dynamics and General Joint Laxity Effect on Disc Pathology

IVD with adjacent vertebras and attached soft tissues are part of functional spinal unit. Panjabi et al., explained the importance of neutral zone being a parameter that correlate well with instability of spinal system [50]. For optimal muscle power generation, good alignment of the bone allow optimal stretch in muscle generating the power required in activities and helps to prevent pathological increase in forces going through the joints. In patients with chronic radiculopathy, disc herniation and severe facet degeneration were associated with altered paraspinal muscle morphology at or below the pathology level [51]. The status of muscle and ligamentous laxity may have an association with prevalence of the lumbar disc disease with significantly higher prevalence of the lumbar disc herniation and also worse clinical and radiological outcomes than patients without general joint laxity with conservative treatment or after surgery [52,53].

### 3.11. Mechanical Load

Lumbar spine overload was associated with work tasks requiring combinations of manual handling of objects and trunk rotation or bending. Those included healthcare, foundry and forest workers, production operators, cabinetmakers, locksmiths and bricklayers [54]. In a study of 4 groups of workers in Korea National Health and Nutrition Examination Survey, lumbar spine degeneration is significantly increased in blue collar and agribusiness and low-level workers [3,55]. Cellular ovine culture studies also showed that the result with mechanical loading with limited nutrition leading to detrimental effects on IVD cells in whole organ culture [56,57]. Chronic and excess exposure to high mechanical load can lead to deleterious effects on IVD, however low rate of loading is important for forced convection aiding in diffusion of nutrients both to normal and degenerated disc. Hence appropriate amount of loading is necessary in preference over complete offloading by bedrest in maintenance of healthy disc [58].

### 3.12. Genetics and Degenerated Disc

Genes played a significant role in disc degeneration. Twin studies shown up to 50–70% of an individual risk of DDD is attributed to genetic disposition [59,60] and Case control studies showed significant familial disposition with DDD [61]. Genes that are postulated to be related to DDD are genes affecting IVD structure, catabolic cyctokines polymorphism and inflammatory cascade cycokine polymorphisms. For genes affecting IVD structure, Aggrecan, COL1, COL9, COL 11, FN, HAPLIN 1, Thrombospondin, Cartilage Intermediate layer protein (CLIP) and Asporin (ASPN) genes has effects in maintenance of IVD structure. The catabolic cytokines polymorphisms are vitamin D receptor gene polymorphisms MMP1, MMP2, MMP3, PARK2, and PSMB9 and anticatabolic tissue inhibitor of metalloproteinases (TIMPs) favor catabolism and can contribute to the degeneration of the IVD [3].

### 3.13. Environmental and Psychosocial Factors Associated with DDD

Smoking, obesity and diabetes mellitus are positively correlated with DDD. Of the 3 factors, smoking is the highest correlations to DDD and their effect is synergistic [62]. ADAMTS5 is the primary aggrecanase found in animal study that mediating smoking-induced disc aggrecanolysis and DDD [63]. Elmasary et al., showed that smoking caused nicotine-mediated down-regulation of cell anabolism affecting the glycosaminoglycan concentration at the cartilage endplate, reducing it up to 65% from normal physiological conditions and reducing of solutes exchange between blood vessels and disc tissue affecting mainly the nucleus pulposus, whose cell density and glycosaminoglycans levels were reduced up to 50% of their normal physiological levels [64,65].

### 3.14. Nutritional Deficiency

Healthy disc required good nutritional supply. Interruption of the nutritional pathway can lead to disc degeneration [2]. Nutrition studies are challenging due to variability of food culture and wide prevalence of DDD as a result to multifactorial factors, the correlation of diet and DDD is weak with insufficient fish and egg consumption [66].

### 3.15. Exercise

Physical exercise is clinically recommended in several guidelines to help in alleviating pain [67] Physical exercise helps in IVD cell proliferation in animal model studies, particularly in moderate to high volume low repetition and frequency exercises [68,69]. It has effect on paraspinal muscle strength and aids in reducing pain and disability [70].

### 3.16. Pathophysiology of Pain in Lumbar Degenerative Disc Disease

After Initial trigger event, several interlinked macro and microscopic changes in the disc leads to development of DDD and pain sensitization. It starts with microscopic damage leading to cytokine secretion which stimulate immune cell migration and further cytokine secretion. The effect is an increase in neutrophils and nerve ingrowth and spinal nerve sensitization leading to lower back pain. The presence of physical irritation by changes in anatomy can lead to further neural compression and neuropathic pain.

### 3.17. Types of Lower Back Pain

There are mainly 2 groups of back pain, specific back pain and non-specific back pain. Specific back pain is divided into 3 subtypes. (A) discogenic back pain, which presents with back and/or leg pain which has no radiological significant compression of nerve roots. (B) nociceptive back pain other than disc such as myofasicial back pain which includes back sprain and back spasms, osteoarthritis back pain which includes synovial joints such as facet joints, vertebral fractures and osteoporosis. (C) Neural leg pain such as radicular pain secondary to nerve root compression Non specific back pain may arise spontaneously without evidence of any anatomical abnormalities, the exact cause of non specific back pain is unknown, biopsychosocial factors play important role in non specific back pain. Centralization occurs when pain becomes chronic and pathological neuronal pathways may form when disc is degenerated [71].

### 3.18. Adjacent Vertebra End Plates and Bodies Stressed Related Pain Response

Previously it was thought that discogenic back pain as its name suggests is predominantly due to disc origin of pain. However more evidence recently suggests that adjacent vertebral end plates play a significant role in discogenic back pain with the main pathologic innervation focus on the end plates region [31,72].

### 3.19. Modic Changes and Pain Simulation

Modic changes (MC) in MRI shown as signal changes in T1w and T2w images often accompany DDD. Modic Changes prevalence is high in LBP patients (43 % median prevalence in a meta-study) compared to only 6 % median prevalence of the asymptomatic population [73]. There is postulation that Modic changes in bone is a sign of end plate damage with persistent stimulus and damage accumulation coupled with various factors leading to symptomatic response from the Modic changes [74]. MC is associated with more frequent and longer duration of LBP [75]. The size of MC is directly correlated with the severity of LBP [76]. Further analysis with disc and bone tissue obtained around IVD and the adjacent MC showed there is plausible cross communication between these 2 tissues with pairwise correlating expression of similar gene and cytokine interaction in disc and adjacent bone marrow with Modic changes [72]. Disc-secreted factors are important in MC pathobiology, a feature that should motivate new treatment approaches that target MC spinal levels on top of targeted DDD. The cause and nature of the communication between the 2 surfaces are unknown. However, Neurotrophic receptors of the tropomyosin receptor kinase (Trk) family were up-regulated in MC disc and bone marrow, it is postulated to be its communication pathway. But typical appearance of both adjacent cephalad and caudal vertebra bodies adjacent to the diseased disc with MC couple with the fact that there is direct correlation of size of MC to severity of DDD showed there is significant correlation and communication between the interphase [77].

### 3.20. Genetics and Its Effect on Pain Perception in Disc Diseases

Catechol-O-methyltransferase, which is encoded by COMT, is a major mammalian enzyme involved in the degradation of catecholamines, it is postulated to be a putative pain sensitivity gene COMT. Important role of COMT gene in the symptomatic lumbar disc herniation is show in susceptibility studies with the influence of genetic variants of COMT gene in the variation in pain after treatment for low back pain [78,79]. There is an interest in developing relevant genetic markers such as COMT to provide useful clinical information in terms of predicting outcome after surgery for patients diagnosed with DDD [80].

### 3.21. Factors Release Inducing Innervation and Neuronal Sensitization

#### 3.21.1. Inflammatory Processes

Inflammatory response plays a key role in induction of hyperalgesia of the disc as experiments show that exposure of nucleus pulposus to annulus fibrosis leads to increase inflammatory cells around the region of exposure [81,82]. The degenerating disc releases growth factors, such as bFGF and TGF-beta1; together with increased levels of tumour necrosis factor-α, interleukin-1β, Nerve Growth Factor, NGF and Brain Derived Neutrophic Factor, BDNF leads to increase production of macrophages and mast cells which might play a key role in the repair of the injured annulus fibrosus and subsequent disc degeneration [83]. These Factors also stimulate neurite growth and calcitonin gene-related peptide expression, both of which were blocked by anti-NGF treatment. In a protein arrays study, it was found that there is increased levels of 20 inflammatory factors, many of which have nociceptive effects; demonstrating that degenerating and painful human IVDs release increased levels of NGF, inflammatory and nociceptive factors ex vivo that induce neuronal plasticity and may actively diffuse to induce neo-innervation and pain in vivo [84]. Other anti-inflammatory cytokines such as IL-1, IL-6, and COX 2 are associated with DDD and pain related to DDD [85]. Systematic Review on genes and biomarkers for pain in DDD shows that five genetic variants; i.e., OPRM1 rs1799971 G allele, COMT rs4680 G allele, MMP1 rs1799750 2G allele, IL1α rs1800587 T allele, IL1RN rs2234677 A allele, were associated with reduced recovery of lower back pain and three biomarkers; i.e., TNFα, IL6 and IFNα, were associated with persistent back pain [86]. In our endoscopic videos analysis, we found the presence of grade 2 and 3 neovascularization associated with all cases which required sinuvertebral and basivertebral nerves ablation. We postulate that neovascularization and inflammation with adhesion formation is associated with pathological neuronal sensitization.

#### 3.21.2. Neuronal Sensitization and Pathologic Innervation of Disc

Mechanical pressure on a normal disc does not result in pain as compared to a diseased disc. There is suggestion of diseased disc sensitization with growth of nerve fibers within the fissures and even lying within the deep layers [87,88]. The nerve fibres often described are sinuvertebral nerve (SVN) and basivertebral nerve (BVN).

#### 3.21.3. Sinuvertebral Nerve

**Figure 2:**
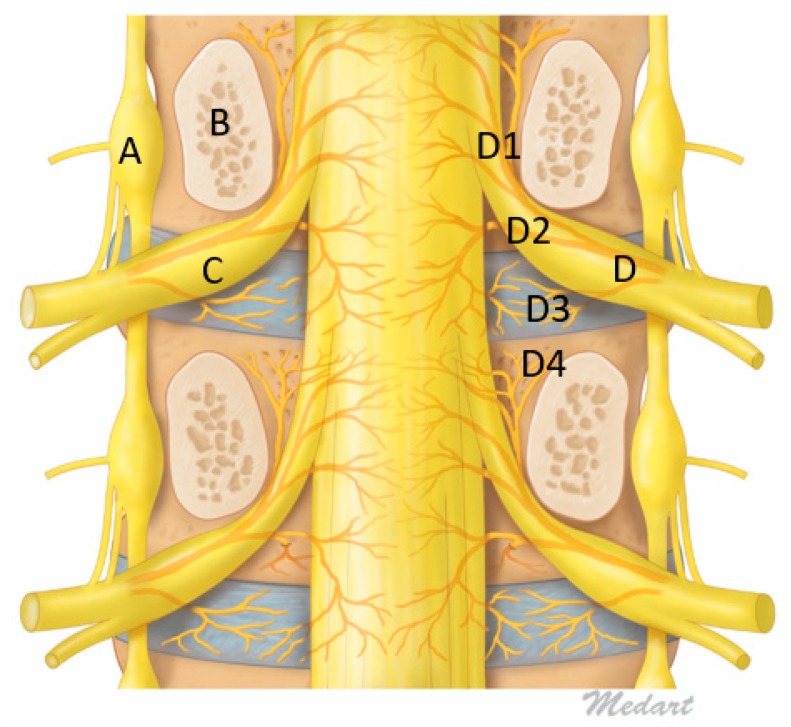
Figure of Coronal Mid Pedicle Cut of Lumbar Spine. A: Sympathetic ganglion, B: Pedicle, C: Dorsal Root Ganglion, D: Sinuvertebral Nerve giving rise to branches D1: Ascending branch which goes intraosseous and give rise to Basivertebral Nerve near the pedicle D4, D2: Descending Branch supplying adjacent to Posterior longitudinal ligament and disc, D3: Direct branches to intervertebral disc.

Sinuvertebral nerve was first described by Dr. Hubert von Luschka in 1850, as a nerve derived from spinal nerve and has connection to sympathetic nervous system with intersegmental anastomoses, now it has been shown that sinuvertebral nerve distribution is extending as far as posterior annulus fibrosus [89,90]. The sinuvertebral nerve is formed by the union of a somatic root from the ventral ramus and an autonomic root provided by the grey ramus. It arises bilaterally from the ventral ramus of each spinal nerve just distal to the dorsal root ganglia, supplying both proprioceptive and nociceptive fibers and joined by grey ramus communicans to provide the sympathetic supply to sinuvertebral nerve. After the union with grey ramus communicans, it takes a recurrent course to re-enter the spinal canal through the intervertebral foramen via the deep anterior intraforaminal ligament, lying alongside the pedicle cephalad to the corresponding disc [89]. Sinuvertebral nerve distribution is controversial whether it is segmented at the spinal nerve of origin level or branching out rostrally and caudally in spinal canal. The opinions is divided on its distribution [8] Various studies show that the nerve divides into superficial and deep networks around the posterior longitudinal ligament with the superficial network predominantly sympathetic and deep network being somatic supplying annulus and posterior longitudinal ligaments [90]. However Nakamura showed that sympathetomy can cause up to 90% loss of sensory fibres to the posterior annulus fibrosus suggesting that both sympathetic and somatic networks are connected [91]. Besides supplying the annulus, the posterior longitudinal ligament, studies showed that sinuvertebral nerve supplies several other anterior spinal structures such as ventral surface of the dura mater extending to the lateral aspects sparing the dorsal surfaces of dura, the periosteum of the vertebrae and the ligaments of facet joints, for the somatic fibers, however it does not supply sensation to the facet joint which is supplied by medial branch of the posterior ramus [7,92]. While the sympathetic fibers also innervate surrounding anterior spinal vasculature in the outer annulus, end plates, vertebral bodies and vertebral marrow (Figure 2).

Since sinuvertebral nerve is both somatic and sympathetic, the information transmitted by sinuvertebral nerve is an important clinical question. Cavanaugh et al., stimulate New Zealand white rabbits were used in series neurophysiologic and neuroanatomic studies. It shows that extensive distribution of small nerve fibers and free nerve endings in the superficial annulus of the disc and small fibers and free nerve endings in adjacent longitudinal ligaments with long lasting excitatory changes after dissection of grey ramus communicans of these rabbits, suggesting that it is more visceral afferents predominant pain pathway in discogenic pain [93].

Various substances and peptides had been implicated as the carrier of neuronal message in the sinuvertebral nerve. Tyrosine vasoactive intestinal polypeptide, substance P and calcitonin gene related peptide are found in nociceptive fibers in both superficial and deep divisions of sinuvertebral nerve. Most of these substances are found in sympathetic nerve transmission peptides, hence supporting previous findings that it has sympathetic nerve supply [94,95,96].

There is controversy on how the sinuvertebral nerve communicate and flow through the lumbar spinal system. Morinaga using retrograde transport markers cholera toxin B and horseradish peroxidase crystals demonstrated retrograde flow from L5/6 to L1/2 in rats [97]. Cavanaugh et al., demonstrated a similar ascending track in sympathetic flow from lower lumbar vertebrae [93].

### 3.22. Discogenic Back Pain and Sinuvertebral Nerves

Sinuvertebral nerve (SVN) is associated with 26–39% of patients with lower back pain [98,99]. It has a variable penetration to annulus in normal and diseased state, penetrating much deeper in diseased state and even as deep as nucleus pulposus [100,101,102]. The penetration is mediated by vascularized granulation formation bringing in the neurotropic factors and facilitating the pathologic penetration of sinuvertebral nerve fibers to deeper disc region [102,103]. The sinuvertebral nerve fibers are denser in the end plates of degenerated disc as compared to normal disc [104]. Studies showed that when sinuvertebral nerve penetrated deep enough to be in contact with nucleus pulposus, various side effects occurs such as increased neural damage, inflammatory cells accumulation and increased capillary permeability and increment of nerve discharge [82,105]. In our experience, which we published in the literature, we find patients with sinuvertebral and basivertebral nerve pain presenting with radicular pattern of pain without any significant radiological and endoscopic evidence of significant irritations [106,107].

#### 3.22.1. Basivertebral Nerve

Basivertebral nerve (BVN) is a paired nerve believed to play an important role in end plate pain nociceptive transmission and are branches from sinuvertebral nerve complex (Figure 2). BVN transmits pain signal from end plate [31,72,74]. BVN are branches of SVN which enters vertebral body through central vascular foramen together with basivertebral vessels and branches around the end plates [12,108]. The transmission substances are found to be substance P, protein S-100, PGP 9.5 and CGRP which are predominantly nociceptive neurotransmitters [84,109,110,111]. BVN density is proportional to the amount of disc damage, creating evidence of its role in chronic lower back pain [31]. As disc degenerates and adjacent end plates showing concordant damage, a convective flow induced during cyclical spinal loading allows cross talk between disc and bone marrow of subchondral bone through the release of inflammatory mediators and an ineffective healing response of progressively increase inflammatory mediators and response generation but little progress in healing due to inherent relentless cycle of degenerative cascade [72,75,112].

#### 3.22.2. Diagnostic and Treatment Procedures

Patient who presents with degenerative disc disease has mechanical pain which is worse on flexion related activities as more compressive stress is placed on the IVD during flexion. The pain may radiate to the bilateral buttock region but unlike radicular pain due to compression of nerve, discogenic pain seldom goes below the level of the knees. The diagnostic gold standard test is provocative discogram which would reproduce the lower back pain that the patient experienced. It involves injection of a contrast into the disc concern and concurrent assessment of the patient’s pain response, it is important to assess the pattern of pain which should be similar to the pattern that patient complains of. A well performed discogram can be more specific than magnetic resonance imaging [107]. Once proven by provocative discogram, a few strategies in treatment had been shown to have variable effectiveness in the literature. Anesthetic (4% xylocaine or 0.75%) bupivacaine mixed with contrast during discogram can give therapeutic effect on top of diagnostic intervention [9]. Other electrothermal based intervention through catheters such as intradiscal electrothermal annuloplasty (IDET), disc fx [113] and transforaminal epidurosopic laser ablation of sinuvertebral nerve with good clinical outcomes [10,114].

A randomized controlled double blind sham controlled multi-center study showed there is improved of significant clinical improvement using radiofrequency ablation of BVN through transpedicular intraosseous probe insertion [12]. The author’s group also treated BVN with epiduroscopic basivertebral nerve laser ablation (TEBLA) with good clinical results [11].

#### 3.22.3. Radiofrequency Ablation

**Figure 3:**
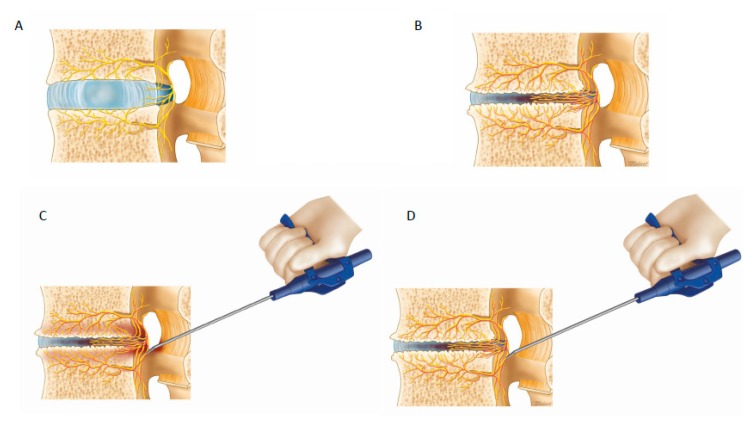
Radiofrequency ablation of the sinuvertebral nerve and basivertebral nerve. In (**A**), normal disc, there is a presence of sinuvertebral nerve branches but no pathological neurotization and no neovascularization. (**B**) Pathological neovascularization and neurotization around the disc with hyperalgesia from signals transmitted by the sinuvertebral nerves and basivertebral nerves. (**C**) When radiofrequency is applied to the pathological hyperalgesic nerve fibers, there is twitching of the patient’s buttock muscles. (**D**) As radiofrequency ablation continues, the pathological neurotized nerve fibers are coagulated and twitching of the buttock muscles stopped in our case series. The patients performed better in clinical outcomes if they had initial buttock twitching and subsequent stoppage of twitching after radiofrequency ablation.

**Figure 4:**
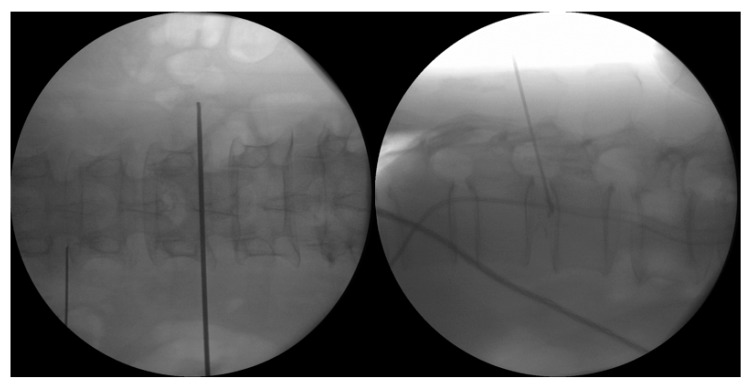
Provocative discogram at L1/2. The left picture shows the landmark of the Antero Posterior view of the L1/2 disc entry point. The right picture shows the lateral view with provocative discogram, the patient had immediate axial back pain and left buttock pain which is similar in nature to her chronic back pain.

Currently in the authors’ practice, we used a protocol which involved first taking a history and physical examination of the patients presenting with lower back pain and radicular leg pain (Figure 3). A typical degenerative disc disease pain would be a patient with no history of cancer or trauma, presenting with chronic lower back pain worse on prolonged standing and activities. Carrying heavy load and bending caused aggravation of pain with radiation to the buttock and radicular pain. After MRI which may showed degenerative disc changes which is classified by Pfirrmann classification with good inter and intra observer agreement [115] and Modic classification of the adjacent vertebral body [39] but no significant compression by Schiaz grading, a decision is made on clinical diagnosis of degenerative disc disease. If symptoms are severe and typical of degenerative disc disease, a provocative discogram (Figure 4) can be done to confirm the diagnosis. Treatment options can be conservative treatment with physiotherapy [6,116], injection, fluoroscopic guided radiofrequency ablation [12,117] and recently to improve accuracy of radiofrequency ablation and ensure safety, we do radiofrequency ablation through endoscopic visualization with transforaminal endoscopic approach [10]. Interlaminar approach can be performed to do radiofrequency ablation of sinuverterbral nerve and basiverterbral nerve as well as shown in this study. The indication is mainly BVN and SVN based discogenic back pain and leg pain with concurrent thickening of ligamentum flavum or L5/S1 discogenic back and leg pain.

In our prospective case series, we applied radiofrequency ablation to sinuvertebral nerve and basivertebral nerve in patients with discogenic back and leg pain without imaging suggestive of significant neural compression. We observed clinical and statistical significant improvement of VAS pain score and ODI score with good to excellent MacNab pain relief score in all except 2 patients who had no twitching of buttock during radiofrequency ablation. And in that 2 patients, the VAS, ODI improvement and MacNab score for pain relief is only fair. The twitching observed is postulated to be due to stimulation of aberrant connections with traversing nerve or exiting nerve root during pathological neurotization of the sinuvertebral and basivertebral nerve. The presence of twitching of buttock is suggestive that we are on the right spot in application of the radiofrequency and there is pathological neurotization. We also used a novel neovascularization grading and found that only patients with grade 2 (presence of neovascularization) and grade 3 (presence of neovascularization and adhesions to neural elements) were found in our patients endoscopic findings. This is an important inferred sign in view that both the sinuvertebral nerve and basivertebral nerve are usually not able to be seen even with endoscopic magnified vision.

Overall, this study was performed in patients with Modic changes in association with pathological increased Mechanical loading component due to degenerative disc disease and its relationship to neovascularization and postulated association with neurotization of the sinuvertebral and basivertebral nerve, successful ablation of these pathological nerves as evidenced by Kim’s twitching which subsided after treatment has a positive clinical outcome, all the selected patients with correct indications and associated Kim’s twitching showed statistically significant improvements in VAS, ODI and MacNab Criteria after application of radiofrequency ablation to sinuvertebral and basivertebral nerve.

### 3.23. Limitations

As this is a novel procedure and concept beginning to gain acceptance in the spine community, the number of cases performed in the study in small with inherent limitations. Procedures performed has confounding factors of approaches (transforaminal compared to interlaminar), amount of bone resection (interlaminar endoscopic lumbar disc approach compared to interlaminar endoscopic unilateral laminotomy with bilateral decompression) with their respective different sizes of scope and working cannula used to perform these procedures. Indications had a mixture of degenerative disc disease with and without degenerated disc protrusion and spinal stenosis without significant facet arthropathy as confounding factors. We attempted to limit the confounding factors by only including patients with back and leg pain without significant neural compression by Schiaz grade. Among the bulging degenerative disc, we selected only degenerated disc protrusion cases which discectomy was not performed and radiofrequency ablation was done on similar specific spots. Radiofrequency ablation is also applied in region of the bulging disc which we believed have connecting fibres to sinuvertebral nerves and basivertebral nerves though these areas of bulging disc.

### 3.24. Summary of Discogenic Back Pain Pathway and the Role Sinuvertebral Nerve and Basivertebral Nerve in Molecular Science

Discogenic back pain is a clinical challenge for scientists and physicians. It encompasses a complex pathway leading to significant disability. It starts with disc injuries leading to disruption of the structure of the disc. Such damages can happen in minor repeated microrepetitive injuries to disc leading to mechanical structural disruption, chemical and inflammatory pathways activation. These activations leads a vicious cycle of destruction of the disc and interacts with the adjacent vertebral bodies which science community postulates that it leads to Modic changes. These chemical and inflammatory reactions can lead to acute back and radicular pain initially which may subside with conservative management. However if the microscopic mechanical instability, chemical and inflammatory reactions continued unabated, neovascularization and pathological neurotization may occur which leads to pathological sensitization of sinuvertebral nerve and basivertebral nerve. Chronic sensitization of these pathological nerves can lead to centralization of pain.

In our opinion, many of the myriad of pain pathways in degenerative disc diseases that have not been resolved so far are likely inter-related problems. Treatment had been targeted at different levels by various physicians, (1) at the disc level directly by various agents to remove parts of diseased disc or pain generators (2) in fusion to stabilize the mechanical instability (3) in oral anti-inflammatory medications epidural steroids taken in acute phase and chronic phase to decrease the chemical and inflammatory reactions to disc injuries, (4) in radiofrequency ablation of sinuvertebral and basivertebral nerves which are the nerves related to the discogenic back pain which is the focus of our study (5) in medications and devices such as spinal cord stimulation to decrease centralization related chronic pain.

Such a wide myriad of solutions to a common clinical problem means that the true solution is still eluding the scientific community, perhaps molecular science can contribute research in the diagnosis and subsequent correct targeted treatment developments which can lead us to finding the solution to this common clinical problem.

Molecular science can play a significant role in the advancement of the mystery in discogenic back pain. First, molecules specific to the chemical and inflammatory response to disc injury would be helpful for diagnosis. Second, Modic changes is closely associated to pathological mechanical loading, many of the studies of discogenic pain, including molecular biology, that had been conducted in the past, have not been successful in finding the linkage between discogenic pain during rest and during mechanical loading in the form of pain pathway. Part of the reason is because mechanical loading dynamic loading response molecules and the pain pathway molecules might be different. If we can find a molecular pathway in detecting pathological mechanical loading leading to Modic changes it would be helpful in diagnosis of discogenic back pain. Third, molecules specific to adhere to neovascularization processes in the region of sinuvertebral and basivertebral nerves or specific to these pathological nerves can be helpful in diagnosis of sinuvertebral and basivertebral nerves related discogenic back pain. Forth, MRI detectable contrast can be tagged to these molecules to home in the area of pathological sinuvertebral and basivertebral nerves for better diagnosis and targeted treatment.

## 4. Materials and Methods

**Figure 5:**
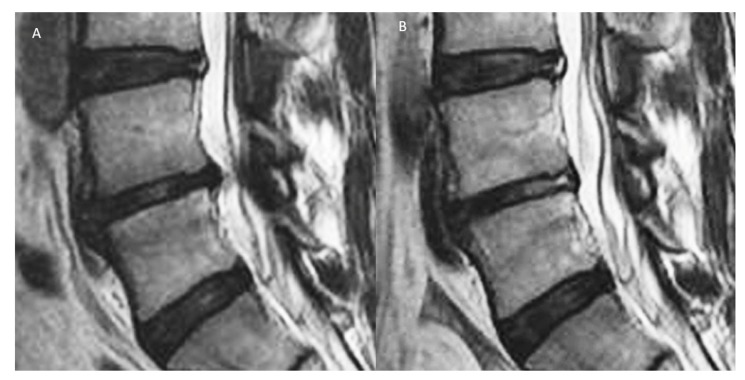
(**A**) Degenerative disc disease with Pfirrmann grade III disc, showing inhomogeneous structure, and an unclear distinction of nucleus and annulus and intermediate signal intensity in T2 image with slightly decreased disc height and disc bulge. (**B**) Radiofrequency ablation of the disc, sinuvertebral nerve and basivertebral nerve showing shrinkage of degenerative disc and there is an increase in the signal of Modic changes in the adjacent vertebra body.

A prospective case series evaluation was performed for patients who had uniportal spinal endoscopic radiofrequency ablation of both basivertebral nerve and sinuvertebral nerve as a treatment for chronic discogenic back pain between 1 June 2016 to 1 June 2018 performed by single senior spine surgeon. We include patients who had chronic discogenic back pain as they met inclusion criteria of (1) clinical presentation back-associated leg pain in a dermatomal distribution of at least 6 months in duration with documented failure of conservative management [106] (2) Magnetic Resonance Imaging (MRI) showing degenerative disc PfirrmannType 3 [118] and above disc changes with or without degenerated disc bulge and Modic Changes of either type 1 and 2 (Figure 5). We included patients without any clear compression of nerve roots based on Schiaz grading in imaging studies, thickened ligamentum flavum is acceptable for the study as long as no clinically significant neural compression with degenerated disc bulge [119]. Preoperative Discography was performed in patients who had no other spinal pathology except axial back pain and non bulging degenerative disc disease as a diagnostic test to confirm concordant discogenic back pain. Intraoperative Discography would be performed if there were other spinal pathologies which warrants surgery such as bulging degenerative disc disease and spinal stenosis with concurrent discogenic back pain from degenerative disc disease. We excluded patients with previous spinal surgery, traumatic fracture, infection and tumor. We excluded patients with predominant non mechanical leg pain, patients with neurogenic claudication. We excluded patients with MRI showing prolapsed uncontained disc or significant facet arthropathy. We also excluded patients who required discectomy or removal of any disc fragment for neural compression during endoscopic spine surgery.

The senior surgeon (HSK) would use interlaminar uniportal full endoscopic approach for patients with associated spinal stenosis and when the disc pathology occurs in L5/S1 level. While transforaminal endoscopic lumbar approach was performed on lumbar one to lumbar five levels without spinal stenosis. In either approach, the procedure would end with sinuvertebral and basivertebral nerve ablation as the final step.

### 4.1. Surgical Technique

In all 3 types of endoscopic surgeries, the procedure consists of 2 parts. First part is to (1) safely access to the spinal canal with or without removing ligamentum flavum and (2) thermal shrinkage of disc protrusion by radiofrequency coagulator. Second is to expose the region of sinuvertebral and basivertebral nerve in order to perform radiofrequency ablation to basivertebral nerve and sinuvertebral nerve.

#### 4.1.1. Interlaminar Endoscopic Lumbar Approach to Disc

**Figure 6:**
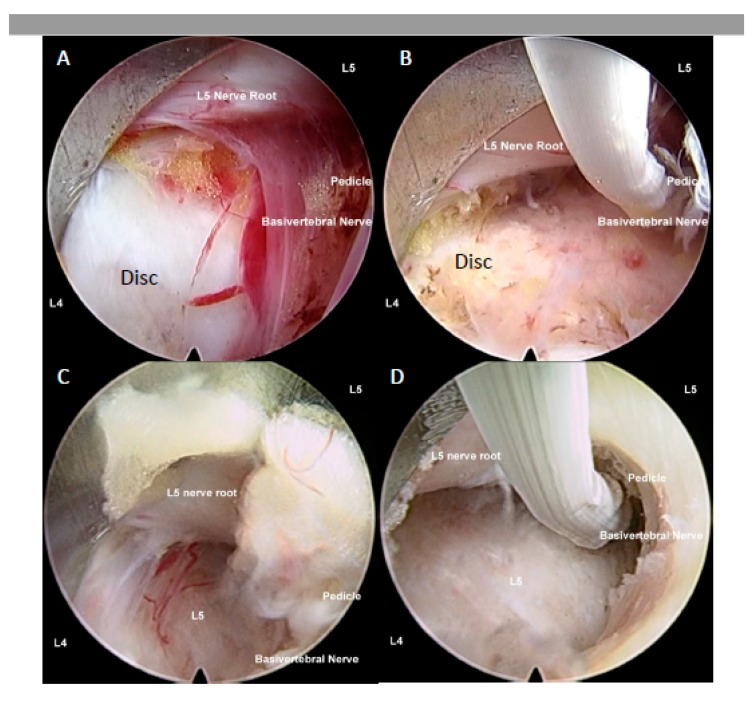
(**A**) Uniportal interlaminar endoscopic approach to disc showing the relationship of the lumbar five (L5) traversing nerve root which is retracted away by the working channel, exposing the disc of L4/5, the basivertebral nerve is located above pedicle of left L5, there is grade 3 neovascularization and inflammatory granulation tissue with adhesion around the basivertebral nerve region. In most circumstances, the basivertebral nerve is too fine to be seen by endoscopic vision. (**B**) Same region in the same patient after radiofrequency was applied to shrink the degenerative disc, and to ablate the pathological neovascularization with underlying inflammatory tissues and the basivertebral nerve, the typical response is twitching of the buttock when the correct location of the basivertebral nerve is ablated. (**C**) Another patient with similar steps in retraction of traversing nerve root and exposing neovascularized tissue and the location of the basivertebral nerve. (**D**) Radiofrequency ablation applied to neovascularized tissue, disc and basivertebral nerve.

Procedure is done under sedated regional or general anesthesia. Patient is placed in prone position. Endoscope is docked on the junction of ipsilateral facet joint and intervertebral disc line on intraoperative fluoroscopy. Serial dilation is done and the TESSYS Endoscopic System (Joimax GmbH, Karlsruhe, Germany) with endoscope of 30° viewing angle, a 7.3 mm outer diameter, a 4.7 mm-diameter working channel, and 251 mm of total length is introduced to docking point. Hemostasis performed and soft tissue dissection done to expose ligamentum flavum. Ligamentum flavum is split using a probe and working channel is pushed into the spinal canal away from the neural elements towards the disc space. Working channel is manipulated with rotation to retract neural elements out of harm’s way. Disc was inspected for any uncontained herniated disc fragments which would be excluded from the study. Degenerated bulging disc which were included in the study would have radiofrequency thermal shrinkage procedure performed on the annulus surface [120,121,122,123] (Figure 6).

#### 4.1.2. Lumbar Endoscopic Unilateral Laminotomy for Bilateral Decompression with Radiofrequency Ablation

Procedure is done under regional or general anesthesia. Patient is placed in prone position. An iLESSYS Delta Endoscopic System (Joimax GmbH, Karlsruhe, Germany) is used for endoscopic work. This system has a working cannula of 13.7 mm outer diameter and 10.2 mm inner diameter. The endoscope has a 10-mm outer diameter and a 6-mm working channel, and a 15-degree view angle. Endoscope is docked on the junction of ipsilateral facet joint and intervertebral disc line on intraoperative fluoroscopy. Using endoscopic diamond burr drills, we drilled just enough lamina to safely remove the thickened flavum so as to safely approach the targeted disc. We routinely switched the endoscope to a 30° viewing angle, a 7.3 mm outer diameter, a 4.7 mm-diameter working channel, and 251 mm of total length for closer examination of the disc and end plates region and to perform radiofrequency ablation to the sinuvertebral and basivertebral nerves [120,121,122,123].

#### 4.1.3. Transforaminal Endoscopic Lumbar Approach for Radiofrequency Ablation

**Figure 7:**
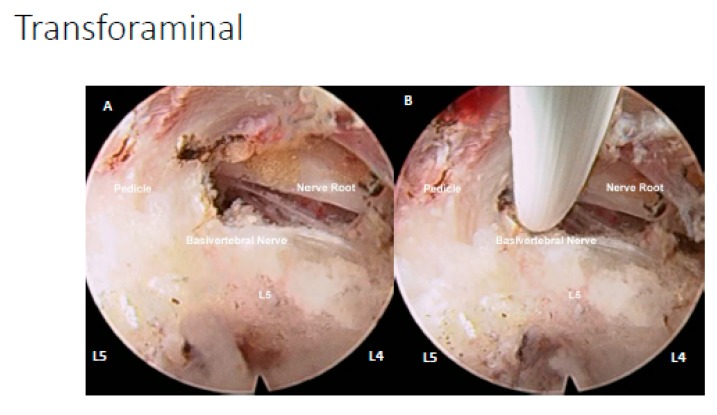
(**A**) Right transforaminal endoscopic approach of L4/5 disc space, under endoscopic view we can assess the traversing nerve root of L5 to be decompressed, L5 pedicle and location of basivertebral nerve above the pedicle are observed. (**B**) Application of radiofrequency ablation on the basivertebral nerve.

Procedure is done under local anesthesia with moderate sedation. Patients were placed in prone position on Wilson Frame [124] (Figure 7). Skin marking is made on the entry point which is in line with intervertebral disc on anteroposterior view of intraoperative fluoroscopy in a slight 5–10° cephalad caudal direction and with 25–35° angle to horizontal in axial plane about 12–14cm from midline of lumbar. The angle of approach varied according to the position of the degenerated disc protrusion Needle is docked at Kambin’s triangle near the intervertebral disc which is marked by space between hypotenuse as exiting nerve root, base as superior border of the inferior vertebra and height as traversing nerve root [125]. We used endoscope of 30° viewing angle, 7.3 mm outer diameter, 4.7 mm-diameter working channel, and 251 mm of total length. We performed mobile outside in technique with drilling of ventral superior articular facet to have better access to disc [124]. Parts of disc were removed to create space in facilitating the approach to the region of sinuvertebral nerve and basivertebral nerve. The region of sinuvetebral nerves is commonly located in the region just below the center of annulus. In order to ablate this area safely under the view, removal of parts of ventral degenerated disc annulus was inevitable. The region of basivertebral nerves is commonly located close to the upper medial part of pedicle and in order to expose this area, removal of some part of ventral annulus is inevitable to safely access the region of basivertebral nerve.

### 4.2. Ablation of Neuropathic Basivertebral Nerve and Sinuvertebral Nerve

In both transforaminal and interlaminar approaches, the final step is performing radiofrequency ablation. A bipolar radiofrequency electrocoagulator (Elliquence, Baldwin, NY, USA) is used in ablation of neuropathic basivertebral nerve around the suprapedicular area (Figure 2 and Figure 3) and sinuvertebral nerve in mid disc region between posterior annulus and posterior longitudinal ligament. This is performed with the working channel protecting the neural elements, making sure it is out of harm’s way. At the area of pathological neurotization of Basivertebral and Sinuvertebral Nerves, there would typically be neovascularization with or without adhesion tissues, which we grade and record accordingly. Then bipolar radiofrequency probe is applied to medial suprapedicular area, we observe for twitching of the buttock when application of bipolar radiofrequency and typically it starts twitching when basivertebral nerve is coagulated. We termed this iatrogenic twitching phenomenal when the neural elements are well protected out of harm’s way by working cannula as Kim’s twitching as it is a sign of the correct targeted treatment area. Once of basivertebral nerve was completely ablated, Kim’s twitching would stop even when radiofrequency applied to the same area which was twitching initially. Sinuvertebral nerve on the other hand is just caudal to mid disc region of the intervertebral disc in the space between the posterior annulus of the disk and the posterior longitudinal ligament (PLL), when radiofrequency is placed on the right region, either buttock of the patient would also have Kim’s twitch accordingly and once sinuvertebral [10].

### 4.3. Data Collection

**Figure 8:**
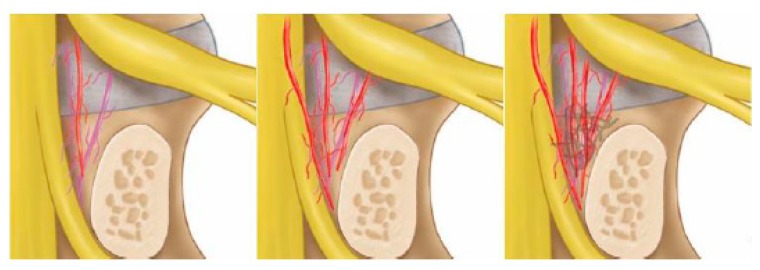
Drawings of the neovascularization grading system. Left picture showing a grade 1 normal appearance with sparse epidural vessels around the disc. The middle picture showed a grade 2 increased neovascularization of epidural vessels with vascularization. The right picture showed grade 3 with increased neovascularization of epidural vessels with vascularization and adhesion on neural tissues.

Basic parameters of age, sex and indication of surgery of the patients are collected, nature of surgery and levels of surgery is documented. For each patient, the following information was collected preoperatively and during follow-up: visual analogue scale (VAS) scores for low-back pain, Oswestry Disability score, and and MacNab’s criteria are used in evaluation of patients’ pain response and disability. Clinical parameters are measured at pre-operative, 1 week, 6months and final follow up in a prospective data collection. The medical files were reviewed for data on intraoperative and postoperative complications including bleeding, infection and revision posterior decompression. Endoscopic videos review were performed to evaluate the presence of intraoperative twitching and grade the degree of neovascularization and adhesion. In this novel grading, Degenerative Spinal Neovascularization Grade. We defined as follows: Grade 1. Normal vascularization, Grade 2. Neovascularization without adhesion, Grade 3. Neovascularization with adhesion (Figure 8).

**Figure 9:**
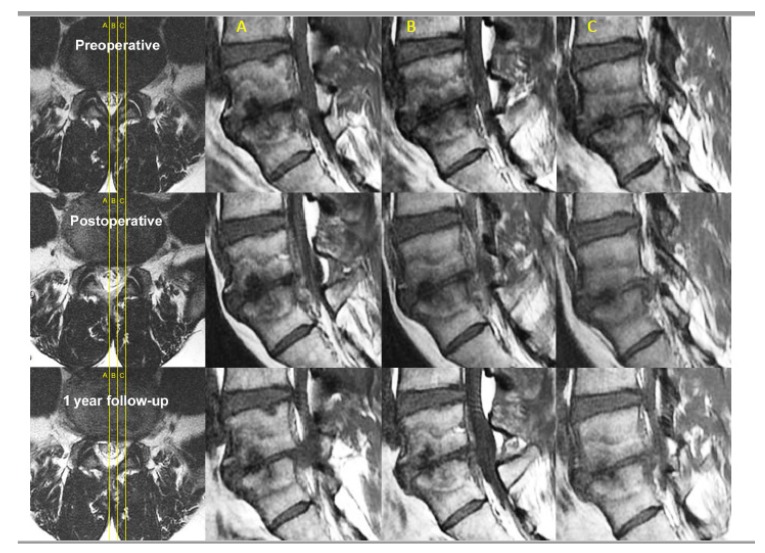
Sagittal cuts across L5/S1 in a patient with spinal canal stenosis, degenerative disc disease of L5/S1 with disc protrusion into the spinal canal and vertebral bodies of L4 and L5, there are Type 1 Modic changes signifying bone marrow edema and inflammation. The corresponding axial and sagittal cuts are labelled (**A**–**C**) accordingly. On axial cuts, we can see preoperative spinal canal stenosis with disc bulge and ligamentum hypertrophy with Schiaz grade A3 with rootlets lying dorsally occupying more than half of the dura sac area. This patient suffered buttock pain and both leg pain especially right leg pain for 5 months. The patient visited the clinic with visual analogue scale (VAS) score of 8 that was aggravated from 3 months ago, especially buttock pain and right leg pain, in spite of the preoperative MRI and spinal stenosis was not severe (Schiaz Grade A3). We can check the severe Modic change (Type 1) in the adjacent vertebrae. The patient had decompressed and radiofrequency ablation to sinuvertebral and basivertebral nerves in MRI postoperative day 1 which had been maintained in a 1-year follow up. There is shrinkage of the disc and less marrow changes in bone edema. The patient’s final follow-up VAS was 1 and returned to work as per normal.

Magnetic Resonance Imaging is done pre-operatively and post-operatively. We observed type of Modic changes, Schiaz [126] grade for degree of spinal stenosis and adequacy of decompression in patients with spinal stenosis and shrinkage of disc in patients who had degenerative disc disease and degenerated disc protrusion with or without other pathologies (Figure 9).

### 4.4. Statistical Analysis

Clinical data was analyzed with SPSS version 18 statistical analysis software (IBM corporation, New York). The continuous variables were expressed as mean and standard deviation (SD). The *paired t* test is used for comparison of pre-operative and post-operative visual analogue scale (VAS), Oswestry Disability Index (ODI) and MacNab’s outcome criteria to assess patient’s outcome at pre-operative, 1 week, 6 months and final follow up. Statistical significance is when *p* value is less than 0.05.

## 5. Conclusions

Pathoanatomy and pathophysiology of degenerative disc disease is a subject of intensive scientific research, the pathological neurotization of sinuvertebral nerve and basivertebral nerve can lead to siginificant pain and disability. Radiofrequency ablation to sinuvertebral and basivertebral nerve is effective in our prospective case series. More investigation should be considered in the treatment of sinuvertebral and basivertebral nerve.

## Figures and Tables

**Table 1:** Background data and results of Patients who underwent Sinuvertebral Nerve and Basivertebral Nerve Radiofrequency Ablation.

Age (Mean/Range)	48	18–71	
Sex (Male/Female)	13	17	
Diagnosis Types			
Degenerative disc disease without protrusion		4	
Spinal Stenosis		12	
Degenerated Disc Protrysion		14	
Follow up duration (Mean/Range)	27	4–120	
Types of Operation		23	
Interlaminar Diac Approach		23	
Interlaminar Stenosis Approach		12	
Transforaminal Approach		6	
Neovascularization Grade		0	
Grade 1		0	
Grade 2		6	
Grade 3		24	
Modic Type			
Type 1		24	
Type 2		6	
Schiaz Grade			
Grade A1		25	
Grade A2		5	
Grade A3		1	
Mean/SD VAS (Pre and Final)	7.4 ± 0.7	1.7 ± 1.5	*p* < 0.05
Mean/SD ODI (Pre and Final)	71.8 ± 1.88	20.8 ± 14.9	*p* < 0.05
MacNab Criteria			
Fair		2	
Good		11	
Excellent		17	

## Abbreviations

IVD	Intervertebral Disc
DDD	Degenerative Disc Disease
ODI	Oswestry Disability Index
VAS	Visual Analogue Scale
AF	Annulus Fibrosus
NP	Nucleus Pulposus
PLL	Posterior Longitudinal Ligament
SVN	Sinuvertebral Nerve
BVN	Basivertebral Nerve
